# Fetal Lead Exposure: Timing Is Everything for Effects

**Published:** 2006-11

**Authors:** Carol Potera

Many countries have set guidelines for levels of environmental lead exposure that are considered safe for children. However, relatively few studies have focused exclusively on the role of prenatal lead exposure on infant neurodevelopment. Indeed, studies conducted in the past 20 years have shown inconsistent results, perhaps because of variability in when prenatal lead was measured (first, second, or third trimester) and in what type of sample (maternal plasma, maternal whole blood, or umbilical cord blood). A comprehensive study published this month is the first to compare such variables **[*EHP* 114: 1730–1735; Hu et al.]**.

From 1997 to 1999, the investigators measured lead levels of 146 pregnant women living in Mexico City. Leaded gasoline was sold in Mexico City until 1997, and bone lead levels in women there are about three times higher than in the United States. The leaching of lead stored in a mother’s bones provides a major source of fetal lead exposure.

The investigators obtained samples of plasma and whole blood during each trimester and umbilical cord blood at delivery. They also tested the neurodevelopment of the children at age 24 months using the Mental Development Index (MDI), which evaluates memory, language, and sensory abilities.

The authors found that lead exposure during the first trimester of pregnancy was more strongly linked to later decreases in the MDI scores than exposure during the latter two trimesters. Moreover, maternal plasma lead was the best predictor of a child’s later neurobehavioral performance because most of the lead in whole blood is attached to red cells and cannot cross the placenta. Each increase of 1 standard deviation unit in plasma lead lowered the MDI score by 3.5 points. Neither maternal levels in the second or third trimester nor cord blood levels impacted MDI scores in as strong a fashion.

The results raise two questions: should lead be routinely measured in the first trimester of pregnancy, and are there ways to reduce fetal exposure? Plasma lead is expensive and difficult to measure, according to the authors, making routine clinical testing impractical. Studies suggest that calcium supplements slow the release of lead from bone. An ongoing clinical trial of pregnant women is assessing the efficacy of this intervention.

## Figures and Tables

**Figure:**
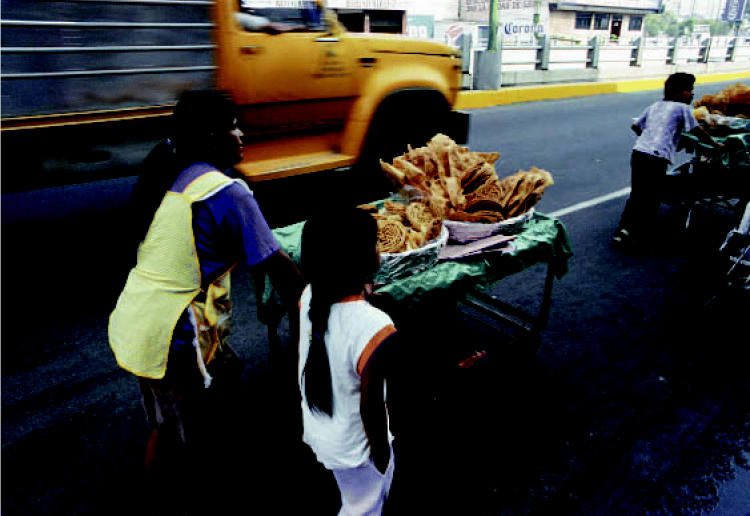
Early threat A study of pregnant women in Mexico City showed that fetal lead exposure during the first trimester had a greater impact on later neurodevelopment than exposure in other trimesters.

